# A Holistic Approach To Oncofertility In A Patient With Rectal Cancer: A Case Report

**DOI:** 10.5935/1518-0557.20220073

**Published:** 2023

**Authors:** Koray Görkem Saçıntı, Elena-Narcisa Predut, Yavuz Emre Şükür, Cem Somer Atabekoğlu

**Affiliations:** 1 Department of Obstetrics and Gynecology, Ankara University School of Medicine, Ankara, Turkey; 2 Faculty of Medicine, University of Medicine and Pharmacy of Craiova, Craiova, Romania

**Keywords:** ovarian tissue cryopreservation, embryo cryopreservation, combined technique, fertility preservation, ovarian transposition

## Abstract

**Objective:**

Cryopreservation techniques are used to preserve fertility before cancer treatment with gonadotoxic agents. Herein, we report a case of fertility preservation involving a 29-year-old G0P0 woman, married for one year, who was referred to our hospital for fertility preservation before starting rectal cancer treatment.

**Case description:**

Ovarian tissue and embryo cryopreservation were performed. Before the procedure, ovarian reserve was evaluated, and antral follicle counts were determined. Laparoscopic ovarian tissue cryopreservation was performed from the left side with a lower antral follicle count. Thus, we were able to keep the number of oocytes obtained in the following controlled ovarian hyperstimulation cycle at the highest level. Subsequently, the right ovary was transposed into the lateral wall of the abdomen under the peritoneum. Conventionally controlled ovarian hyperstimulation was initiated on the first postoperative day, depending on the menstrual cycle phase. Intracytoplasmic sperm injection was performed on four mature oocytes obtained, and one embryo was cryopreserved. Controlled ovarian hyperstimulation was initiated on the first postoperative day, and the process was repeated on the seventh postoperative day, yielding a total of seven viable embryos for cryopreservation.

**Conclusions:**

There is usually only one chance of controlled ovarian hyperstimulation in patients requiring a fertility-sparing approach due to malignancy. In the combined technique, performing ovarian tissue resection from the ovary with a lower number of antral follicles can keep the number of oocytes at the highest level in the following controlled ovarian hyperstimulation cycle.

## INTRODUCTION

Fertility preservation has become a necessity for oncologic patients who undergo chemotherapy and radiotherapy, since cancer treatment may reduce or eliminate the chances of achieving pregnancy and impair ovarian endocrine function. Pelvic radiation in cancer treatment comes with many side effects, with the reduction of ovarian follicles as the most concerning one. The lethal dose 50 (LD50) for the human oocyte was estimated to be less than 2 Gy (Wallace *et al*., 2003). The impact of chemotherapy on ovarian impairment has been studied, and three main mechanisms have been considered: primordial follicle DNA damage followed by apoptosis, growth of dormant follicles and therefore apoptosis, and the induction of ovarian vascular damage, all of them leading to ovarian reserve decline (Sonigo *et al*., 2019). In order to prevent premature ovarian insufficiency or imminent ovarian failure, patients scheduled to receive gonadotoxic therapy must be informed about treatment risks, and a strategy for fertility preservation must be developed. Given the fact that the current techniques used in oncofertility cannot ensure success in all cases, a holistic approach should be observed, with combined treatment preferred to maximize the chance of pregnancy.

Herein, we report a case of fertility preservation involving a 29-year-old patient diagnosed with rectal cancer scheduled to undergo chemotherapy and radiotherapy. We used a novel approach for the combined technique, adding ovarian transposition and an experimental method based on antral follicle count when choosing the ovary for wedge resection.

## CASE DESCRIPTION

A 29-year-old G0P0 woman, married for one year, was referred to our hospital for fertility preservation before gonadotoxic treatment for rectal cancer. The treatment plan included chemotherapy and radiotherapy. In order to design the fertility preservation plan, we considered the starting time of cancer treatment and decided there was enough time for controlled ovarian hyperstimulation; hence we selected the holistic approach to increase the chance of a successful live birth. Ovarian reserve was determined by counting the number of follicles in both ovaries. We chose the left ovary for a wedge resection since it was the one with the lower number of antral follicles (Table 1). Right ovarian transposition was indicated to protect the ovary from pelvic radiation.

**Table 1:** Data of the patient who chose the holistic approach for fertility preservation.

Age (y)	AMH	Right AFC	Left AFC	OTC	OT	No. of oocytes collected	MII oocytes cryopreserved	Embryos cryopreserved
28	5.2	8	5	Left	Right	18	13	7

AMH: anti-Müllerian hormone (ng/mL); AFC: antral follicle count; OTC: ovarian tissue cryopreservation; and OT: ovarian transposition.

**Figure 1:**
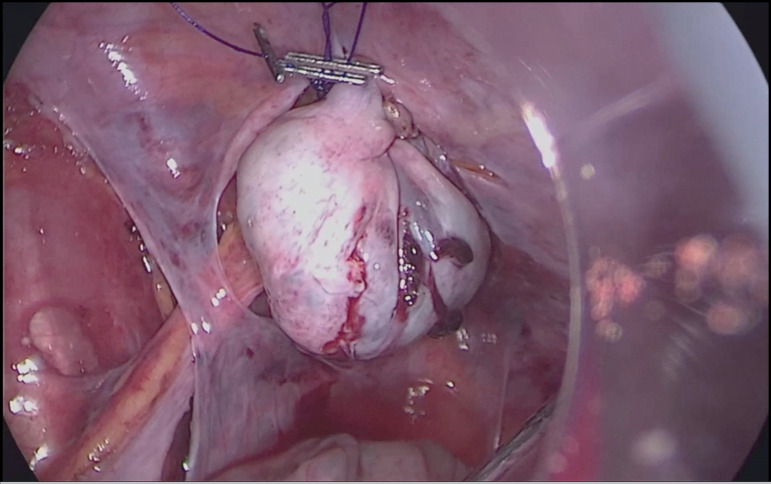
Laparoscopic right ovarian transposition for fertility preservation.

A laparoscopic procedure was performed following preoperative preparation. The left ovary was resected and sent to the Reproductive Health Center for cryopreservation. In the right iliac fossa, necrosis of the fallopian tube was observed, and right salpingectomy was performed. The utero-ovarian ligament was cauterized and separated in order to free the right ovary. We ensured that the transposition distance for the ovary was at least 3 cm from the upper edge of the radiation field. Incision of the peritoneum facilitated the mobilization of the ovary. Ovarian transposition was performed by passing the right ovary under the peritoneum and fixing it to the lateral abdominal wall with a Prolene suture (Ethicon, Inc., Somerville, NJ) (Figure 1). Extra care was taken to protect ovarian blood supply. The gap in the broad ligament was closed, and the distal side of the ovary was marked with a hemoclip. The procedure ended once hemostasis was achieved and the skin incision closed. The patient had no postoperative complications.

**Figure 2:**
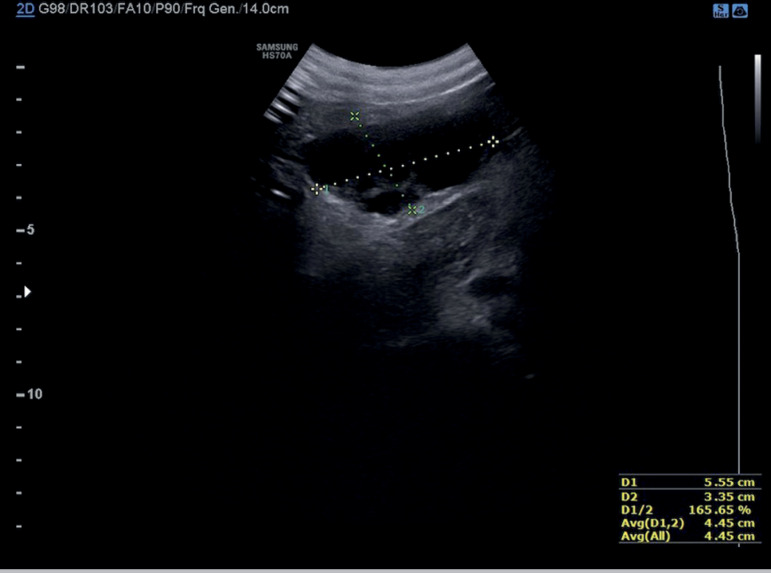
Transabdominal ultrasonography of the transposed right ovary.

On the first postoperative day, controlled ovarian hyperstimulation was initiated for further oocyte pick-up. The dose for gonadotropin needed was calculated based on patient age and ovarian reserve (225 IU). On the 6th day of stimulation, a GnRH antagonist (Cetrotide TM; Serono, Inc.) was added, and when follicles reached a diameter of 18mm, recombinant hCG was administered to complete oocyte maturation (Figure 2). When the two most prominent follicles reached 18 mm in diameter, final oocyte maturation was achieved with recombinant hCG (r-hCG, choriogonadotropin alfa-Ovidrel; Serono, Inc.). Transvaginal oocyte retrieval was performed 35.5 hours later, and four mature oocytes inseminated by intracytoplasmic sperm injection (ICSI), yielding one embryo viable for cryopreservation. The entire process was repeated nine days later, and a new embryo cryopreservation cycle was initiated. This time two oocytes were retrieved via the abdominal and seven via the transvaginal approach. Intracytoplasmic sperm injection was performed on nine mature oocytes, from which six embryos reached Day 3 and were eligible for cryopreservation. Seven embryos were cryopreserved. The patient is still undergoing cancer treatment and has not sought fertilization therapy yet.

## DISCUSSION

The ideal approach involves oocyte or embryo cryopreservation in post-pubertal patients with sufficient time for controlled ovarian hyperstimulation before the administration of gonadotoxic therapy. However, there is usually only one chance of controlled ovarian hyperstimulation in patients requiring a fertility-sparing approach due to malignancy. In these patients, ovarian tissue freezing is considered an appropriate method as a second approach to increase the chances of fertility with a limited number of oocytes to be used when necessary. In addition, ovarian transposition is a valid approach for patients scheduled for pelvic radiotherapy.

According to Rienzi et al., 20 oocytes are required to achieve a live birth, although this number is usually a problem for cancer patients (Rienzi *et al*., 2012). A model to predict the likelihood of live births from elective oocyte cryopreservation was created as a guide to estimate the number of oocytes needed to achieve a live birth based on the age of the female patient: 20 frozen oocytes for women aged 34, 37, or 42 years yield chances of live birth of 90%, 75%, and 37%, respectively (Goldman *et al*., 2017). Controlled hyperstimulation of the follicles and oocyte maturation takes time. However, prompt introduction of cancer treatment is crucial and delays cannot be tolerated. Besides, oocyte quality is frequently poor in oncological patients (Dolmans *et al*., 2014). This statement particularly emphasizes the need to adopt a combined approach for our patients.

As a solution for the effects of radiotherapy on patient fertility, laparoscopic repositioning of the ovaries to areas outside the radiation field is recommended, mainly for women aged 35 years and younger, with a 60.3% ovarian survival rate at five years post-radiotherapy versus 0% in the control group (Hoekman *et al*., 2019). When comparing the different pelvic radiation methods, ovarian transposition followed by brachytherapy gives the best results, ranging from 63.6% to 100% efficiency in preserving ovarian function versus other methods used in pelvic radiotherapy that can drop the odds of success of ovarian transposition by 20% (Hoekman *et al*., 2018). In current literature, ovarian tissue cryopreservation before controlled ovarian hyperstimulation and oocyte pick-up does not affect the quality or the number of oocytes; for this reason, combining the two techniques can only increase the potential of fertility preservation (Rienzi *et al*., 2012).

Regarding ovarian tissue cryopreservation, the likelihood of restoring reproductive function of the ovaries after ovarian tissue transplantation with frozen tissue is 45% (Sheshpari *et al*., 2019). Ovarian tissue cryopreservation is performed as the ultimate backup plan to attempt live birth despite its relatively low success rate. The data on combined techniques and their effect on long-term follow-up studies and case reports is limited, thus keeping this method as an experimental one. Gathered information from international multi-center reports should reassure reproductive health physicians that the combined technique can be successfully used.

A combined technique should be preferred to preserve fertility to increase treatment efficiency, since none of the available procedures can guarantee pregnancy when used individually; therefore, we must maximize our patient’s chances to produce children after gonadotoxic treatment by combining the available methods, meticulously taking into consideration personal characteristics like marital status, cancer therapy plan, patient age, and type of cancer.
